# Glycidol Fatty Acid Ester and 3-Monochloropropane-1,2-Diol Fatty Acid Ester in Commercially Prepared Foods

**DOI:** 10.3390/foods10122905

**Published:** 2021-11-24

**Authors:** Yuko Shimamura, Ryo Inagaki, Minami Oike, Beibei Dong, Wan Gong, Shuichi Masuda

**Affiliations:** School of Food and Nutritional Sciences, University of Shizuoka, 52-1 Yada, Suruga-ku, Shizuoka 422-8526, Japan; shimamura@u-shizuoka-ken.ac.jp (Y.S.); s16601@u-shizuoka-ken.ac.jp (R.I.); foodhygn@u-shizuoka-ken.ac.jp (M.O.); gp1534@u-shizuoka-ken.ac.jp (B.D.); gp2078@u-shizuoka-ken.ac.jp (W.G.)

**Keywords:** glycidol, glycidyl fatty acid esters, 3-monochloropropane-1,2-diol esters, processed foods

## Abstract

Glycidyl fatty acid esters (GEs), which are the main pollutant in processed oils, are potential mutagens or carcinogens. 3-Monochloropropane-1,2-diol fatty acid esters (3-MCPDEs) are also well-known food processing contaminants. 3-MCPDEs are believed to be a precursor to GEs in foodstuffs. In vivo, lipase breaks down the phosphate ester of GEs and 3-MCPDEs to produce glycidol and 3-MCPD, respectively, which are genotoxic carcinogens. Thus, it is important to determine human exposure to GEs and 3-MCPDEs through foodstuffs. There are only reports on the amount of GE and 3-MCPDE in cooking oils and cooked foods. The content in multiple types of foods that are actually on the market was not clarified. In this study, 48 commercially prepared foods were analyzed to identify other sources of exposure to GE and 3-MCPDE. All of them contained relatively high amounts of GEs and 3-MCPDEs. The correlation between GEs and 3-MCPDEs in individual foods was examined. There was a correlation between the amounts of GEs and 3-MCPDEs in the food products (*r* = 0.422, *p* < 0.005). This is the first report on the content in multiple types of commercially prepared foods that are actually on the market was clarified.

## 1. Introduction

Glycidyl fatty acid esters (GEs) are process contaminants. They are produced from a deodorization process performed under high-temperature conditions during the production of edible oils. Diacylglycerol (DAG) oil was found to contain considerably higher GE levels compared with other commercial edible oils and was discontinued. The formation of GEs can occur during cooking with all refined edible oils and processed foods made with these oils [1]. GEs are degraded in vivo by the action of lipase to produce glycidol (2,3-epoxy-1-propanol), which contains a reactive epoxy site in its structure [2]. Glycidol was reported for its genotoxicity and carcinogenicity [3,4] and is considered a rodent carcinogen according to the National Toxicology Program study. The International Agency for Research on Cancer (IARC) has defined glycidol as a Group 2A carcinogen (“probably carcinogenic to humans”). Therefore, human exposure is a concern because glycidol is ingested through a diet containing GEs.

Based on the life expectancy of human erythrocytes, hemoglobin adducts can indicate exposure levels to target chemicals over the last 120 days. Therefore, hemoglobin adducts are used as markers for evaluating long-term exposure to various reactive substances. *N*-(2,3-dihydroxypropyl) valine (diHOPrVal) has been reported to be a hemoglobin adduct of glycidol and represents a useful exposure marker [5]. Honda et al. measured diHOPrVal levels in subjects exposed before and after discontinuing the use of DAG oil. As a result, there were no significant differences associated with DAG oil use [6]. This suggests that we are routinely exposed to GEs from sources other than DAG oil. We discovered that GEs were contained in grilled meat and fish in as study examining new sources of GEs exposure [7]. In particular, higher concentrations of GEs were detected in meat cooked on charcoal that is heated at high temperatures. Landin et al. (2000) reported that rats fed with a fried diet for about 70 days showed an approximate 50% increase in diHOPrVal levels [8]. Therefore, the heating of food was suggested as a possible source of GEs. However, information on the GE content in commercially available prepared foods is limited.

3-Monochloropropane-1,2-diol (3-MCPD) fatty acid esters (3-MCPDEs) are also well-known food processing contaminants. They are degraded in vivo by the activity of lipase to form 3-MCPD [2]. IARC has defined 3-MCPD as a Group 2B carcinogen (“possible human carcinogens”). The experimental animals fed with high doses of 3-MCPD for extended periods show renal hyperplasia and tumors of the reproductive organs [9]. 3-MCPDEs have been detected in refined palm oil [10], oil used for various heat-processed foods [11], and infant formula [12]. The potential nephrotoxicity of 3-MCPDEs from 3-MCPD raises concerns about exposure levels to these food contaminants. 3-MCPDEs are believed to be a precursor to GEs in foodstuffs [3]. The commercially prepared foods high in 3-MCPDEs may also be high in GEs.

Although there are reports on the content of GE and 3-MCPDE in experimentally cooked foods [13,14], the content in multiple types of foods on the market was not clarified. On the other hand, the possibility that glycidol produced from GEs is a genotoxic carcinogen cannot be ruled out. Therefore, it is required to clarify the actual content of GEs and its precursor 3-MCPDE in prepared foods. In the present study, we measured the amounts of GEs and 3-MCPDEs in commercially prepared foods to identify their new sources other than cooking oil and grilled foods.

## 2. Materials and Methods

### 2.1. Chemicals

The standard materials of glycidyl palmitate (C16:0-GE, purity 98.0%), glycidyl stearate (C18:0-GE, purity 98.0%), glycidyl oleate (C18:1-GE, purity 98.0%), glycidyl linoleate (C18:2-GE, purity 90.0%), glycidyl linolenate (C18:3-GE, purity 85.0%), 3-MCPD di-palmitate (purity 95.0%), 3-MCPD di-stearate (purity 98.0%), 3-MCPD di-oleate (purity 98.0%), 3-MCPD dilinoleate (purity 98.0%), and 3-MCPD di-linolenate (purity 98.0%) were purchased from Wako Chemicals (Osaka, Japan). The standard solutions of the 3-MCPDEs and GEs (0.01–10 ppm) were diluted with methanol (Kanto Chemical Co., Inc., Tokyo, Japan)/2-propanol (Wako Chemicals) (1:1 *v*/*v*) and used for liquid chromatography-tandem mass spectrometry (LC-MS/MS). All other reagents used in this study were of analytical grade.

### 2.2. Measurement of GE-Related Substances in Various Prepared Foods

#### 2.2.1. Sampling

Food samples were divided into 8 categories as follows: 5 samples of instant noodles, 6 samples of fried chicken, 7 samples of fried confectionery (potato and wheat), 6 samples of fried bread, 3 samples of hamburger steak, 2 samples of grilled saury, 3 samples of canned grilled chicken, 2 samples of mayonnaise, and 6 samples of dressing. In addition, 8 other cooked frozen foods were evaluated (Table 1, product 41: spring rolls; 42: fried pork cutlet; 43: fried noodles; 44: ginger grilled pork; 45: fried mashed potato; 46: chicken kebab; 47: fried rice; and 48: squid tempura, which is covered in batter and fried in oil). Sampling was performed in retail stores in Japan. Samples were stored as recommended by the manufacturer and analyzed within the post-collection expiration date.

#### 2.2.2. Purification of Prepared Foods

Samples were ground with a mixer and freeze-dried. After the addition of 150 mL of diethyl ether to 10 g of dry sample, oil extraction was performed by Soxhlet extraction for 8 h. For liquid samples (30–60 g), approximately 100 mL each of diethyl ether and purified water was added. The diethyl ether layer was extracted by shaking for 30 min. Diethyl ether extract was evaporated with a vacuum concentrator to collect the crude oils. Then, 1 g of crude oil was diluted with t-butyl methyl ether/ethyl acetate (4:1, *v*/*v*) solution to a total volume of 10 mL. A reverse-phase SPE column (Sep-Pak Vac RC C18 cartridge 500 mg, Waters, Milford, MA, USA) was preconditioned with 4 mL of methanol and then 1.0 mL of the oil sample solution was loaded onto the column. Then, 2 mL of separate aliquots of methanol were applied to the column and eluted. The same procedure was repeated 3 times. A total of 6 mL methanol solution was purged by a nitrogen stream. Next, the normal phase SPE (Sep-Pak Vac RC Silica cartridge 500 mg, Waters) column was preconditioned with 4 mL of n-hexane/ethyl acetate (95:5 *v*/*v*). The residue after the purge was dissolved in 2 mL of n-hexane/ethyl acetate (95:5 *v*/*v*) and loaded onto the column. The same procedure was repeated 3 times. After elution, 6 mL of eluate was dried by nitrogen stream. The dried residue was dissolved in 0.5 mL of methanol/2-propanol (1:1 *v*/*v*). The solutions were used for the analysis of GEs and 3-MCPDEs by LC-MS/MS.

#### 2.2.3. Measurement of GEs and 3-MCPDEs in Prepared Foods by LC-MS/MS

GEs and 3-MCPDEs were measured using a UPLC-tandem mass spectrometer (Waters Corporation, Milford, MA, USA) and a XevoTQ-D instrument (Waters Corporation) with an electrospray ionization (ESI) source. An L-Column 2 ODS (2 μm, 2.1 × 100 mm) (Chemicals Evaluation and Research Institute, Tokyo, Japan) was used. The mobile phase solvent A: 3 mM ammonium acetate in methanol:water = 98:2, and B: 3 mM ammonium acetate in 2-propanol:water = 98:2. The gradient elution program was as follows: 0–3 min, isocratic elution 100% A; 3–9 min, 20–100% A; 9–11 min, 0% A; 11–12 min, return to initial conditions. The flow rate and the injection volume were 0.2 mL/min and 2 μL, respectively. MS conditions were set as follows: declustering potential 65 V, entrance potential 9.5 V, collision energy 30 V, nebulizer gas 80 arbitrary units (au), heater gas 80 au, curtain gas 20 au, ion spray voltage 5500 V, and vaporizing temperature 300 °C. LC-MS/MS acquisition parameters (MRM mode) for GEs and 3-MCPDEs are shown in Table 2. The limit of detection (LOD) and the limit of quantification (LOQ) were set using a signal-to-noise (S/N) ratio (LOD:3, LOQ:10).

### 2.3. Relationship between the amount of GE and 3-MCPDE in Prepared Foods

To determine the relationship between the GE and 3-MCPDE content in prepared foods, the Pearson correlation coefficient (*r*) and the probability *p* value were calculated in Microsoft Excel 2016 (Microsoft, Redmond, WA, USA).

## 3. Results

### 3.1. Measurement of GE-Related Substances in Prepared Foods

#### 3.1.1. Amount of GE in Prepared Foods

**Figure 1:**
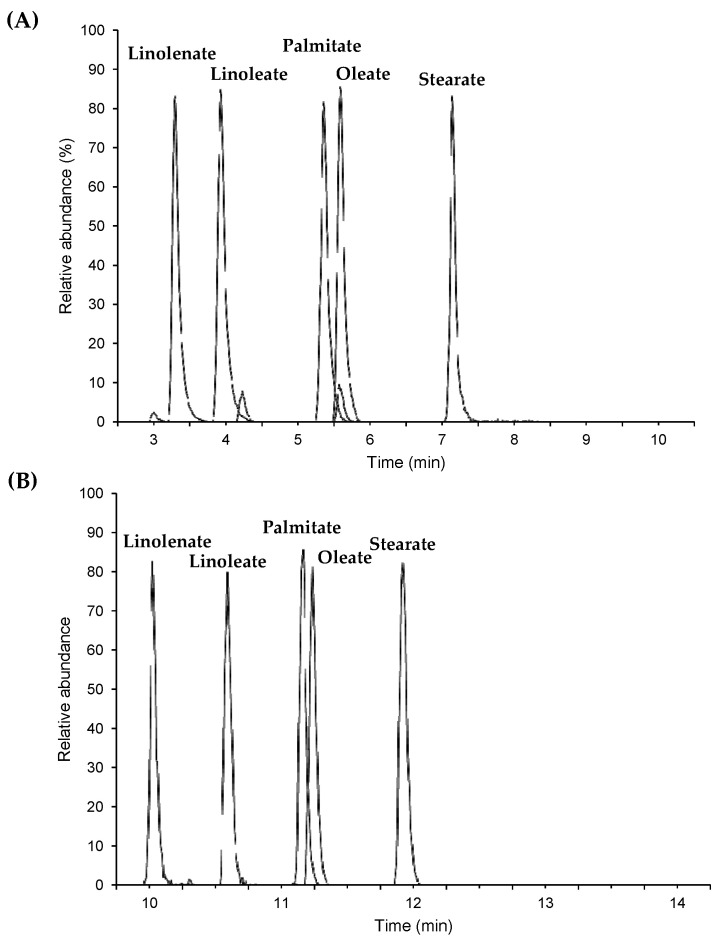
Typical LC-MS/MS chromatogram of GEs and 3-MCPDEs standard sample. (**A**) GEs standard sample (1 ppm) and (**B**) 3-MCPDEs standard sample (1 ppm).

The content of GE was measured using LC-MS/MS from 48 prepared food samples purchased in retail stores in Japan. The list and principal characteristics of the foods are presented in Table 1, which summarizes the amount and type of oils/fats used by the producers and listed on the package label. Figure 1 shows a chromatogram of a standard mixture of GEs (A) and 3-MCPDEs (B). As shown in Figure 1, all standard sample mixtures could be separated.

The total GE contents are shown in Table 3 and Table S1. The total GE contents were high in instant noodles, fried chicken, and fried confectionery (257–579 ng/g). GEs were detected in instant noodles and fried chicken, with the highest amount of oleate fatty acid, followed by palmitate. In fried confectionery, GEs were detected with the highest amount of oleate fatty acids, followed by linoleate. Although GEs in fried bread, mayonnaise, and dressing were less than that of instant noodles, fried chicken, and fried confectionery. In fried bread, mayonnaise, and dressing, GEs were detected with the highest amount of oleate fatty acids, followed by linoleate.

Hamburger steak, grilled saury, and canned grilled chicken, which were not fried and used cooking oil, had relatively little GE content. In hamburger steak, GEs were detected with the highest amount of oleate fatty acids, followed by palmitate. In grilled saury and canned grilled chicken, GEs were detected with the highest amount of palmitate fatty acid, followed by stearate. Except for hamburger steak and grilled saury, the amount of glycidyl oleate was the highest. Other cooked frozen foods contained high GE oil content in fried products (Product 42: fried pork cutlet, 259.3 ng/g; 43: fried noodles, 293.9 ng/g; and 48: squid tempura, 100.4 ng/g) (Table 1 and Table S1).

#### 3.1.2. Amount of 3-MCPDE in Prepared Foods

The content of 3-MCPDE in prepared foods was measured using LC-MS/MS (Table 4 and Table S2). The total 3-MCPDE contents were high in instant noodles and fried confectionery (19.0–72.6 ng/g). 3-MCPDEs were detected in instant noodles and fried confectionery, with the highest amount of oleate fatty acid, followed by palmitate. 3-MCPDEs in fried chicken, fried bread, mayonnaise, and dressing were less than instant noodles and fried confectionery. Although 3-MCPDEs in fried chicken, fried bread, mayonnaise, and dressing were less than instant noodles and fried confectionery, 3-MCPDEs were detected at 2.8–12.5 ng/g. Hamburger steak, grilled saury, and canned grilled chicken, which were not fried, had relatively little 3-MCPDE content. The contents of oleate and palmitate fatty acid were also high in fried chicken, fried bread, mayonnaise and dressing, hamburger steak, and canned grilled chicken. In grilled saury, 3-MCPDEs were all detected as 3-MCPD oleate diesters. Throughout, the amount of 3-MCPD oleate diester was the highest. Except for instant noodles, 3-MCPD linoleate and linoleate diester were not detected. Other cooked frozen foods had a high 3-MCPDE content, such as fried rice (product 47: 48.4 ng/g) and squid tempura (product 48: 54.8 ng/g) (Table 4 and Table S2).

### 3.2. Relationship between GE and 3-MCPDE Contents in Foods

**Figure 2:**
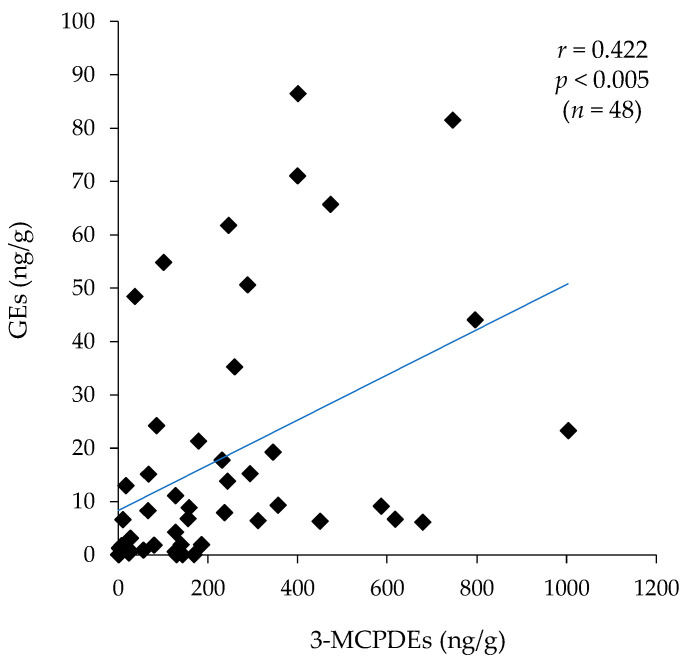
Plot of the amounts of GEs and 3-MCPDEs in individual food products.

Since 3-MCPDE is believed to be the precursor of GE [3], there may be a correlation between the contents of GEs and 3-MCPDEs. Therefore, the correlation between GEs and 3-MCPDEs in individual food was examined. As shown in Table 5, there was no correlation between GEs and 3-MCPDs in instant noodles, fried chicken, and fried confectionery, which had high total contents. Figure 2 shows a plot summarizing the amount of GE and 3-MCPDE in all prepared foods (*n* = 48). There was a correlation between the amounts of GEs and 3-MCPDEs in the food products (*r* = 0.422, *p* < 0.005).

## 4. Discussion

It has been reported that GEs and 3-MCPDEs are produced by the high-temperature heating of oil [15]. Our previous study revealed that GEs are produced by cooking meat patties [7]. To examine the potential for glycidol hemoglobin adduct production from foods other than cooking oil and cooked meat, GE and 3-MCPDE contents were measured in 48 prepared food samples purchased in retail stores in Japan.

The total GE content was high in instant noodles, fried chicken, and fried confectionery (257–579 ng/g) (Table 3). Other cooked frozen foods contained high GE oil content in fried products (42: fried pork cutlet, 259.3 ng/g; 43: fried noodles, 293.9 ng/g; and 48: squid tempura, 100.4 ng/g; Table 1 and Table S1). Although GE contents in fried bread, mayonnaise, and dressing were less than those of instant noodles, fried chicken, and fried confectionery, GEs were detected at 94–292 ng/g (Table 3). Since dressings and mayonnaise are not heated, GEs are considered to be derived from the vegetable oil used. Hamburger steak, grilled saury, and canned grilled chicken, which were not fried and used cooking oil, had relatively little GE content (Table 3). The amount of GE in refined vegetable oils (*n* = 20) at retail stores ranged from 0 to 44.3 ng/g [16]. It was reported that when potato chips are fried in batch mode at 160 or 180 °C for 100 min a day for 5 days, GEs in the frying oil increased [17]. In addition, fried oil is often reused and is heated for a long time, so high concentrations of GE may further accumulate in processed food fried with oil. The amount of GE originally contained in edible oil may vary with heating. In our previous study, we demonstrated that large amounts of GE are produced when meat or fish were cooked at high temperatures [7]. In particular, it became clear that approximately 10 times more GE was detected after heating with charcoal fire compared with gas, suggesting that cooking conditions affect the amount of GE produced in animal-based foods, such as livestock meat and fish. Therefore, it is possible that GE is contained in various processed foods as well as cooking oils and cooked animal foods, and that GE exposure from these foods affects the amount of glycidol hemoglobin adducts.

The formation of 3-MCPDEs occurs at temperatures as low as 160–200 °C [18,19]. The total 3-MCPDE content was high in instant noodles and fried confectionery (19.0–72.6 ng/g) (Table 4). Other cooked frozen foods exhibited high 3-MCPDE content, such as fried rice (product 47: 48.4 ng/g) and squid tempura (product 48: 54.8 ng/g) (Table S2). Although 3-MCPDE contents in fried chicken, fried bread, mayonnaise, and dressing were less than those of instant noodles, fried chicken, and fried confectionery, 3-MCPDEs were detected at 2.8–12.5 ng/g (Table 4). These foods with high 3-MCPDE contents are prepared at high heating temperatures (e.g., 160–200 °C). 3-MCPDEs were detected at a lower concentration compared with GEs [20]. Hamburger steak, grilled saury, and canned grilled chicken, which were not fried, had relatively little 3-MCPDE content (Table 4). These results also support the results of previous studies. In addition, 3-MCPDEs have been reported to decrease after prolonged heating at 160 and 180 °C [21]. Additionally, in this study, we considered that 3-MCPDEs in instant noodles, fried chicken, fried confectionery, and fried bread were decomposed by heat during the cooking process. Since dressing and mayonnaise are not heated during manufacturing, the 3-MCPDE content is considered to be derived from the edible oil used for preparation.

3-MCPDEs form primarily from the reaction between chlorine containing-compounds and acylglycerols, such as triacylglycerol (TAG), DAG, and monoacylglycerol (MAG). GE is formed primarily from DAG and does not require chlorinated compounds [22]. GE formation begins at about 200 °C, becomes more significant at temperatures >230 °C, and increases exponentially as the temperature rises. When DAG exceeds 3–4% of total lipids, such as palm oil with high free fatty acid, the GE formation increases [23]. The formation of 3-MCPDEs occurs at low temperatures (160–200 °C), and formation does not increase at higher temperatures [18,19]. A strong correlation between the MCPDE and the peroxide value indicates that changes in the MCPDE may be associated with oil oxidation [13]. When the contents of GE and 3-MCPDE were examined with French fries, the contents fluctuated in the order of sodium chloride concentration < frying time < frying temperature [14]. Instant noodles are usually steamed fried foods made from flour, water, and salt (sodium chloride). Molded potato chips contained in fried confectionery also contain salt. It was suggested that oil oxidation and presence of salt in instant noodles and fried confectionery had a great influence on the production of 3-MCPDEs.

Because of the different mechanisms of GE and 3-MCPDE formation, it has generally been considered that there is generally no relationship between the relative levels of 3-MCPDEs and GEs in individual oil samples. Figure 2 show a plot summarizing the amounts of GEs and 3-MCPDEs in the food products (*n* = 48). There was a correlation between the amounts of GEs and 3-MCPDEs in these food products (*r* = 0.422, *p* < 0.005). Many of these food products use palm oil, and palm oil contains a high concentration of DAG ranging from 4% to 12% (about 6.5% on average) [23,24,25]. When cooking temperature is around 200 °C, both GEs and 3-MCPDEs may be generated from DAG oil.

The results of this study suggest that we are routinely exposed to glycidol fatty acid ester-related compounds from various foods, and that exposure affects the level of hemoglobin adduct of glycidol (DiHOPrVal), which is an indicator of glycidol. Aasa et al. reported the glycidol intake for children calculated from the levels of the DiHOPrVal in blood samples [26]. Monien et al. measured DiHOPrVal in maternal blood and cord blood [27]. From these reports, it is believed that the level of DiHOPrVal is affected by dietary food habits.

This is the first report where the content in multiple types of commercially prepared foods on the market was clarified. In the future, it will be necessary to identify other sources of GE exposure, to determine the relationship between food intake and the formation of glycidol hemoglobin adducts, and to perform a more detailed risk assessment of glycidol in humans.

## Figures and Tables

**Table 2:** LC-MS/MS acquisition parameters in multiple reaction monitoring (MRM) mode for GEs and 3-MCPDEs.

GEs	Polarity [ESI]	Retention Time (min)	Precursor Ion (*m/z*)	Product Ion (*m/z*)	Regression Equation (*r*^2^)	LOD (ng/mL)	LOQ (ng/mL)	Recovery Rate (%)
Stearate	[M + H]^+^	7.21	358.61	341.61	y = 226,529x + 1326.5 (0.9999)	1.1	3.7	80.8
Oleate	[M + H]^+^	5.75	341.61	339.59	y = 321,321x + 3932.5 (0.9978)	0.8	2.6	84.2
Linoleate	[M + H]^+^	4.34	352.57	335.55	y = 484,213x + 4361.7 (0.9998)	0.4	1.6	81.4
Linolenate	[M + H]^+^	3.46	354.61	337.58	y = 534,380x + 5780.8 (0.9987)	0.4	1.7	88.7
Palmitate	[M + H]^+^	5.42	330.55	313.53	y = 208,483x + 1805.4 (0.9993)	1.2	4.1	86.9
**3-MCPDEs**								
Stearate	[M + NH_4_]^+^	11.90	660.87	359.58	y = 119,599x − 112.42 (0.9997)	0.2	0.6	93.0
Oleate	[M + NH_4_]^+^	11.34	656.80	357.55	y = 61,877x + 123.48 (1.0000)	0.4	1.2	89.7
Linoleate	[M + NH_4_]^+^	10.65	648.74	355.56	y = 9268.6x + 16.009 (0.9998)	14.6	48.6	91.0
Linolenate	[M + NH_4_]^+^	10.08	652.77	355.52	y = 1319.3x − 8.991 (0.9983)	2.2	7.5	95.0
Palmitate	[M + NH_4_]^+^	11.20	604.41	331.33	y = 188,718x + 1559.5 (0.9998)	0.2	0.5	81.2

**Table 1:** Analyzed food samples and their basic characteristics.

Foodstuff	Product	Total Fat (g/100 g)	Oil Used
Instant noodles	1	21.1	: Palm oil
	2	16.5	: Lard, not given (vegetable oil)
	3	19.7	: Palm oil
	4	17.5	: Palm oil
	5	23.0	: Lard, not given (vegetable oil)
Fried chicken	6	5.0 g/product	: Not given
	7	10.3	: Palm oil
	8	15.1	: Palm oil
	9	12.0	: Palm oil, soybean oil
	10	23.8	: Not given
	11	11.9	: Soybean oil
Fried confectionery (potato)	12	36.0	: Not given (vegetable oil)
	13	26.8	: Not given (vegetable oil)
	14	36.0	: Not given (vegetable oil)
Fried confectionery (wheat)	15	18.1	: Rice oil, shortening
	16	16.7	: Rice oil, shortening
	17	16.0	: Rice oil, rapeseed oil
	18	29.1	: Not given (vegetable oil)
Fried bread	19	20.9 g/product	: Not given (vegetable oil), shortening
	20	14.4 g/product	: Not given (vegetable oil), emulsified oil, shortening, margarine
	21	10.8 g/product	: Not given (vegetable oil), emulsified oil, shortening, margarine
	22	7.4 g/product	: Not given (vegetable oil), emulsified oil, shortening, margarine
	23	22.3	: Not given (vegetable oil)
	24	13.7 g/product	: Processed oil, margarine
Hamburger steak	25	11.0	: Corn oil, palm oil, rapeseed oil
	26	11.8	: Beef tallow
	27	5.8	: Not given (vegetable oil)
Grilled saury	28	12.6	: Not included
	29	16.0	: Not included
Canned grilled chicken	30	9.1	: Not included
	31	10.6	: Not included
	32	9.3	: Not included
Mayonnaise	33	74.7	: Not given (vegetable oil)
	34	73.3	: Rapeseed oil, soybean oil, corn oil
Dressing	35	38.0	: Not given (vegetable oil)
	36	36.0	: Not given (vegetable oil)
	37	19.3	: Rapeseed oil, olive oil
	38	28.7	: Not given (vegetable oil)
	39	25.3	: Not given (vegetable oil)
	40	28.0	: Rapeseed oil, sesame oil
Other cooked frozen foods	41	19.5	: Palm oil, soybean oil, processed oil, powdered oil, lard
	42	17.7	: Rapeseed oil, palm oil
	43	14.1	: Palm oil, shortening
	44	18.2	: Not given (vegetable oil)
	45	26.7	: Rapeseed oil, lard
	46	8.2	: Not included
	47	8.4	: Rapeseed oil, rice oil, lard
	48	14.0	: Safflower oil, palm oil, rapeseed oil

**Table 3:** Concentration of GEs in food samples from eight food categories.

Foodstuff	Total of GEs (ng/g)	GEs (ng/g)				
		Stearate	Oleate	Linoleate	Linolenate	Palmitate
Instant noodles (*n* = 5)	384 ± 95	19.4 ± 4.6	203 ± 49	50 ± 10	1.9 ± 0.4	111 ± 30
Fried chicken (*n* = 6)	418 ± 161	18.5 ± 6.5	232 ± 95	75 ± 22	4.4 ± 1.0	89 ± 41
Fried confectionery (*n* = 7)	418 ± 90	13.0 ± 6.5	211 ± 63	120 ± 19	4.7 ± 0.5	70 ± 26
Fried bread (*n* = 6)	118 ± 24	3.1 ± 2.1	73 ± 18	24 ± 6.0	1.1 ± 0.2	17 ± 7.5
Hamburger steak (*n* = 3)	42 ± 41	2.2 ± 2.0	22 ± 0.2	5.4 ± 0.1	0.7 ± 0.7	11 ± 10
Grilled saury (*n* = 2)	1.6 ± 0.9	0.3 ± 0.2	0.2 ± 0.2	N.D.	0.09 ± 0.09	1.0 ± 0.6
Canned grilled chicken (*n* = 3)	32.3 ± 12	0.05 ± 0.03	17 ± 5.4	5.2 ± 3.4	0.24 ± 0.13	9.8 ± 3.6
Mayonnaise and dressing (*n* = 8)	253 ± 39	2.0 ± 0.5	139 ± 23	87 ± 24	17 ± 3.9	8.0 ± 2.6
Other cooked frozen foods (*n* = 8)	109 ± 102	4.7 ± 4.1	61 ± 59	19 ± 17	1.3 ± 1.0	24 ± 23

The value indicates average ± standard error (SE). N.D.: Not detected.

**Table 4:** Concentration of 3-MCPDEs in food samples from eight food categories.

Foodstuff	Total of 3-MCPDEs (ng/g)	3-MCPDEs (ng/g)				
		Stearate	Oleate	Linoleate	Linolenate	Palmitate
Instant noodles (*n* = 5)	59 ± 14	0.2 ± 0.1	30 ± 8.2	N.D.	0.9 ± 0.9	28 ± 4.4
Fried chicken (*n* = 6)	10 ± 3.0	0.03 ± 0.03	6.8 ± 1.5	N.D.	N.D.	2.6 ± 1.9
Fried confectionery (*n* = 7)	30 ± 11	N.D.	22 ± 7.5	N.D.	N.D.	7.6 ± 3.7
Fried bread (*n* = 6)	6.6 ± 3.8	0.01 ± 0.01	1.8 ± 1.3	N.D.	N.D.	4.7 ± 3.0
Hamburger steak (*n* = 3)	0.6 ± 0.3	N.D.	0.2 ± 0.2	N.D.	N.D.	0.4 ± 0.2
Grilled saury (*n* = 2)	0.08 ± 0.08	N.D.	0.08 ± 0.08	N.D.	N.D.	N.D.
Canned grilled chicken (*n* = 3)	4.7 ± 4.1	N.D.	3.6 ± 3.0	N.D.	N.D.	1.2 ± 1.1
Mayonnaise and dressing (*n* = 8)	9.1 ± 2.3	0.1 ± 0.08	8.6 ± 2.2	N.D.	N.D.	0.4 ± 0.2
Other cooked frozen foods (*n* = 8)	24 ± 20	0.2 ± 0.4	13 ± 8.9	3.5 ± 9.1	4.3 ± 9.5	2.4 ± 3.0

The value indicates average ± standard error (SE). N.D.: Not detected.

**Table 5:** Correlation between GEs and 3-MCPDEs in prepared foods using Pearson’s correlation coefficient.

Foodstuff	*r*	*p*-Value ^a^
Instant noodles (*n* = 5)	0.624	0.261
Fried chicken (*n* = 6)	0.751	0.085
Fried confectionery (*n* = 7)	0.391	0.386
Fried bread (*n* = 6)	0.070	0.895
Hamburger steak (*n* = 3)	0.004	N.D.
Grilled saury (*n* = 2)	N.D.	N.D.
Canned grilled chicken (*n* = 3)	0.611	N.D.
Mayonnaise and dressing (*n* = 8)	0.221	0.678
Other cooked frozen foods (*n* = 8)	0.168	0.692
Total (*n* = 48)	0.422	0.003

^a^ *p*-value is for the corresponding correlations. N.D.: Not detected.

## Data Availability

The data presented in this work are available in the insert article.

## Supplementary Materials

The following are available online at https://www.mdpi.com/article/10.3390/foods10122905/s1, Table S1: Concentration of GEs in individual food samples, Table S2: Concentration of 3-MCPDEs in individual food samples.
