# Supplementary material for: Glycidol Fatty Acid Ester and 3-Monochloropropane-1,2-Diol Fatty Acid Ester in Commercially Prepared Foods
Source: Foods. 2021 Nov 24;10(12):2905. doi: 10.3390/foods10122905 (PMC8700650; doi:10.3390/foods10122905)
Supplement: Supplementary file 1 [file foods-10-02905-s001.zip › foods-1460699-supplementary.pdf]

Table S1. Concentration of GEs in individual food samples

Foodstuff	Total fat (g/100 g)	Total of GEs (ng/g)	Stearate	Oleate	Linoleate	Linolenate	Palmitate
Instant noodles	21.1	745.9	37.3	391.4	89.2	3.4	224.7
	16.5	400.7	18.8	210.4	50.9	2.2	118.5
	19.7	288.3	14.6	153.5	39.6	1.8	78.8
	17.5	245.4	12.8	130.6	37.8	0.7	63.5
	23.0	243.5	13.3	129.5	31.3	1.4	68.0
Fried chicken	5.0 g/product	1003.4	34.2	556.2	153.7	6.1	253.2
	10.3	678.4	36.2	392.9	104.2	3.0	142.1
	15.1	586.2	26.4	352.7	86.3	3.4	117.4
	12.0	157.9	11.5	41.7	78.0	8.2	18.5
	23.8	65.9	1.1	41.6	18.9	4.3	N.D.
	11.9	22.1	1.4	5.6	11.5	1.6	2.1
Fried confectionery (potato)	36.0	796.3	37.7	426.9	175.3	5.6	150.8
	26.8	473.8	13.2	263.0	103.7	3.9	89.9
	36.0	399.7	36.2	15.6	170.8	6.4	170.7
Fried confectionery (wheat)	18.1	356.7	0.2	188.2	126.0	4.5	37.8
	16.7	155.3	2.0	91.4	58.6	2.5	0.8
	16.0	127.1	N.D.	68.4	53.5	4.4	0.9
	29.1	617.7	N.D.	423.8	149.2	5.8	38.9
Fried bread	20.9 g/product	178.2	0.4	94.9	41.4	1.7	39.9
	14.4 g/product	169.0	N.D.	126.6	38.5	1.8	2.0
	10.8 g/product	143.2	12.9	76.4	14.5	1.1	38.3
	7.4 g/product	129.2	N.D.	98.6	27.8	0.9	1.8
	22.3	67.4	5.4	34.7	9.6	N.D.	17.7
	13.7 g/product	27.1	N.D.	10.1	14.8	1.3	1.0

N.D.: Not detected.

Table S1. Continued

Foodstuff	Total fat (g/100 g)	Total of GEs (ng/g)	Stearate	Oleate	Linoleate	Linolenate	Palmitate
Hamburger steak	11.0	125.9	6.1	65.7	16.3	2.1	35.8
	11.8	2.4	0.4	0.7	0.3	0.2	0.9
	5.8	N.D.	N.D.	N.D.	N.D.	N.D.	N.D.
Grilled saury	12.6	2.5	0.5	0.5	N.D.	N.D.	1.5
	16.0	0.7	0.1	N.D.	0.2	N.D.	0.4
Canned grilled chicken	9.1	56.1	0.1	26.7	11.9	0.5	16.9
	10.6	23.5	N.D.	14.9	1.2	0.1	7.3
	9.3	16.3	N.D.	8.4	2.6	0.1	5.3
Mayonnaise	74.7	449.7	1.2	206.1	199.6	40.8	1.9
	73.3	345.5	N.D.	117.8	183.2	22.8	21.7
Dressing	38.0	311.9	4.9	240.6	43.4	12.7	10.3
	36.0	236.6	1.6	136.1	76.1	21.2	1.7
	19.3	230.8	2.7	184.3	30.4	9.1	4.3
	28.7	185.0	2.2	93.0	65.4	9.8	14.5
	25.3	139.8	2.1	57.1	67.3	12.3	1.1
	28.0	127.8	1.7	77.9	31.6	7.7	8.9
Other cooked frozen foods	19.5	84.4	5.3	50.3	10.1	0.5	18.2
	17.7	259.3	9.4	152.3	44.2	3.4	50.0
	14.1	293.9	12.2	164.4	48.2	1.3	67.8
	18.2	7.3	N.D.	4.3	1.6	N.D.	1.5
	26.7	10.2	N.D.	3.6	5.3	1.3	N.D.
	8.2	79.3	4.1	35.6	10.0	0.7	28.9
	8.4	36.8	1.4	20.3	10.3	1.0	3.8
	14.0	100.4	5.1	55.2	18.1	1.8	20.3

N.D.: Not detected.

Table S2. Concentration of 3-MCPDEs in individual food samples

Foodstuff	Total fat (g/100 g)	Total of 3-MCPDEs (ng/g)	Stearate	Oleate	Linoleate	Linolenate	Palmitate
Instant noodles	21.1	86.4	0.5	44.6	N.D.	4.5	36.9
	16.5	81.4	0.1	44.6	N.D.	N.D.	36.8
	19.7	61.8	N.D.	33.7	N.D.	N.D.	28.1
	17.5	50.6	0.4	27.4	N.D.	N.D.	22.8
	23.0	13.8	0.1	N.D.	N.D.	N.D.	13.8
Fried chicken	5.0 g/product	23.3	N.D.	11.5	N.D.	N.D.	11.8
	10.3	9.1	0.2	5.1	N.D.	N.D.	3.8
	15.1	8.8	N.D.	8.8	N.D.	N.D.	N.D.
	12.0	8.3	N.D.	8.3	N.D.	N.D.	N.D.
	23.8	6.1	N.D.	6.1	N.D.	N.D.	N.D.
	11.9	1.1	N.D.	0.9	N.D.	N.D.	0.2
Fried confectionery (potato)	36.0	71.0	N.D.	50.8	N.D.	N.D.	20.3
	26.8	65.8	N.D.	45.2	N.D.	N.D.	20.5
	36.0	44.0	N.D.	31.3	N.D.	N.D.	12.7
Fried confectionery (wheat)	18.1	9.3	N.D.	9.3	N.D.	N.D.	N.D.
	16.7	6.8	N.D.	6.8	N.D.	N.D.	N.D.
	16.0	6.7	N.D.	6.7	N.D.	N.D.	N.D.
	29.1	4.3	N.D.	4.3	N.D.	N.D.	N.D.
Fried bread	20.9 g/product	21.3	N.D.	7.9	N.D.	N.D.	13.3
	14.4 g/product	15.1	N.D.	N.D.	N.D.	N.D.	15.1
	10.8 g/product	3.1	N.D.	3.1	N.D.	N.D.	N.D.
	7.4 g/product	0.1	0.1	N.D.	N.D.	N.D.	N.D.
	22.3	N.D.	N.D.	N.D.	N.D.	N.D.	N.D.
	13.7 g/product	N.D.	N.D.	N.D.	N.D.	N.D.	N.D.

N.D.: Not detected.

Table S2. Continued

Foodstuff	Total fat (g/100 g)	Total of 3-MCPDEs (ng/g)	Stearate	Oleate	Linoleate	Linolenate	Palmitate
Hamburger steak	11.0	1.2	N.D.	0.7	N.D.	N.D.	0.5
	11.8	0.6	N.D.	N.D.	N.D.	N.D.	0.6
	5.8	N.D.	N.D.	N.D.	N.D.	N.D.	N.D.
Grilled saury	12.6	0.2	N.D.	0.2	N.D.	N.D.	N.D.
	16.0	N.D.	N.D.	N.D.	N.D.	N.D.	N.D.
Canned grilled chicken	9.1	13.0	N.D.	9.6	N.D.	N.D.	3.4
	10.6	0.9	N.D.	0.8	N.D.	N.D.	0.1
	9.3	0.4	N.D.	0.4	N.D.	N.D.	N.D.
Mayonnaise	74.7	19.3	N.D.	18.4	N.D.	N.D.	0.8
	73.3	17.7	0.6	17.1	N.D.	N.D.	N.D.
Dressing	38.0	11.1	N.D.	9.6	N.D.	N.D.	1.5
	36.0	7.9	N.D.	7.9	N.D.	N.D.	N.D.
	19.3	6.4	0.2	5.5	N.D.	N.D.	0.7
	28.7	6.3	N.D.	6.3	N.D.	N.D.	N.D.
	25.3	1.9	N.D.	1.9	N.D.	N.D.	N.D.
	28.0	1.9	N.D.	1.9	N.D.	N.D.	N.D.
Other cooked frozen foods	19.5	24.2	1.2	16.0	N.D.	N.D.	7.0
	17.7	35.2	N.D.	27.6	N.D.	N.D.	7.6
	14.1	15.2	N.D.	15.2	N.D.	N.D.	N.D.
	18.2	1.7	0.1	1.2	N.D.	N.D.	0.3
	26.7	6.6	N.D.	6.4	N.D.	N.D.	0.2
	8.2	1.8	N.D.	1.2	N.D.	N.D.	0.6
	8.4	48.4	0.1	14.9	27.6	5.8	N.D.
	14.0	54.8	0.4	21.7	N.D.	28.9	3.8

N.D.: Not detected.
